# Circulating and broncho-alveolar interleukin-6 in relation to body temperature in an experimental model of bovine *Chlamydia psittaci* infection

**DOI:** 10.1371/journal.pone.0189321

**Published:** 2017-12-27

**Authors:** Annette Prohl, Carola H. Ostermann, Christoph D. Rummel, Joachim Roth, Petra Reinhold

**Affiliations:** 1 Institute of Molecular Pathogenesis at ‘Friedrich-Loeffler-Institut’ (Federal Research Institute for Animal Health), Jena, Germany; 2 Institute of Veterinary Physiology and Biochemistry, Justus-Liebig-University Giessen, Germany; Instituto Butantan, BRAZIL

## Abstract

In rodent models of experimentally induced fever, the important role of interleukin-6 (IL-6) as a circulating endogenous pyrogen is well established. Studies employing larger animal species and real infections are scarce. Therefore, we assessed bioactive IL-6 in peripheral blood and in broncho-alveolar lavage fluid (BALF) of calves after intra-bronchial inoculation with vital *Chlamydia psittaci* (*Cp*), with inactivated *Cp*, or with BGM cells. Only calves inoculated with vital *Cp* developed fever (peak at 2–3 days after challenge) and significantly increased IL-6 activity. Controls inoculated with either inactivated *Cp* or BGM cells also expressed increased bioactive IL-6, but no fever developed. Activity of IL-6 in BALF was significantly higher compared to blood serum. This experimental model of *Cp* infection revealed no apparent relation between IL-6 in blood and body temperature, but did reveal a relation between IL-6 and other markers of inflammation in BALF. We conclude that a local inflammatory response in the lungs of infected calves caused fever, which developed by mechanisms including other mediators besides IL-6.

## Introduction

Inflammation occurs in vascularized tissue in response to infection, damage, or immune reactions. The function of an inflammatory response is to destroy or deactivate the initiating irritant. Numerous types of cell release soluble mediators when activated in damaged or infected tissue. Should the inflammatory response exceed a given strength, measurable amounts of these mediators enter the circulatory system and are distributed via the blood to different organs. This results in a complex array of systemic reactions, which are collectively termed the acute-phase response (APR; for details and reviews see: [1, 2]). Many components of the APR are triggered by cytokines released upon the infectious or non-infectious inflammatory stimulation. Interleukin-6 (IL-6) is a major mediator of physiological, haematological and immunological responses which occur during the APR, particularly the regulation of synthesis of hepatic acute-phase proteins [3].

Fever is the most prominent brain-controlled component of the APR. Thus extensive research has focused on the role of IL-6 as a critical or necessary endogenous pyrogen. The following sets of observations support a function for IL-6 as an important mediator of the febrile response. (i) In the experimental model of lipopolysaccharide (LPS)-induced fever, there is a good correlation between circulating IL-6 and febrile changes in body temperature [4]. (ii) Neutralization of LPS-induced bioactive IL-6 has been shown to reduce fever in rats [5–7]. (iii) Mice with global deletion of the IL-6 encoding gene [8, 9] and mice with a deletion of the IL-6 receptor on brain endothelial cells [10] show an attenuated febrile response upon systemic injections of LPS.

There are also arguments against a necessary role for circulating IL-6 in the manifestation of fever. (i) Peripherally administered IL-6 alone has poor pyrogenic capacities [5, 6, 11]. (ii) When fever is induced in rats by injecting replicating *E*. *coli* bacteria, a febrile body temperature is produced at time intervals which lack increased levels of IL-6 in the blood [12, 13]. (iii) Fever induction pathways are distinctly employed depending on the induction of a predominant systemic or localized inflammatory response with a pronounced formation of cytokines only at the site of the inflammatory insult [5, 14, 15]. (iv) There is evidence for critical roles of other inflammatory mediators in the induction of fever, namely prostaglandins or complement fragments, at least in some experimental fever models [16, 17].

It should be noted that the vast majority of experimental fever studies are performed in rats or mice and that few studies aim to mimic natural infections. To fill this gap, we used a bovine model of a respiratory *Chlamydia psittaci* (*Cp*) infection that has been previously characterized with respect to clinical outcome, respiratory dysfunctions, acid-base alterations, cellular and humoral host response, and pathological lung lesions [18–21]. Exploiting this large animal model, the current study was undertaken to assess IL-6 activity in blood and broncho-alveolar lavage fluid (BALF) of healthy calves and in calves exposed to the vital pathogen compared to inoculation of the inactivated pathogen or cell culture medium, respectively. Results reveal that, at least in bovines, IL-6 reaction occurs locally and systemically in a highly sensitive way to both infectious and non-infectious stimuli, and even in the absence of fever or any clinical sign of indisposition.

## Animals, materials, and methods

### Legal conformity and ethics statement

This study was carried out in strict accordance with the German Animal Welfare Act. The protocol was approved by the Committee on the Ethics of Animal Experiments and the Protection of Animals of the State of Thuringia, Germany (“Thüringer Landesamt für Verbraucherschutz”, Bad Langensalza, Germany; Permit Numbers: 04-002/07 and 04-004/11). All experiments were performed at biosafety level 2 under supervision of the authorized institutional Agent for Animal Protection. Bronchoscopy was strictly performed under general anaesthesia. Throughout the study, every effort was made to minimize suffering.

### Animals

In this prospective and controlled study, 49 conventionally raised calves (Holstein-Friesian, male) were included. Animals originated from a single farm without any history of *Chlamydia*-associated health problems. Before the study, the herd of origin was regularly checked for the presence of *Chlamydia* spp. by the OIE and National Reference Laboratory for Chlamydiosis. Calves were purchased at the age of 14 to 30 days weighing between 46.2 and 77.6 kg. After a quarantine period of at least 21 days and confirmation of a clinically healthy status, animals were included in the study. Exclusion of co-infections was performed as described previously [18]. Throughout the entire study, animals were reared under standardized conditions (room climate: 18-20°C, rel. humidity: 60-65%) and in accordance with international guidelines for animal welfare. Groups were housed separately. Nutrition included commercial milk replacers and coarse meal. Water and hay were supplied *ad libitum*.

### Study design

The study ran from one week prior to inoculation until five weeks after inoculation. The 49 calves included in this study were divided into four groups. Nineteen calves were inoculated with vital *Cp* previously propagated in buffalo green monkey kidney (BGM) cell culture using standard procedures. The dose of intrabronchial inoculation was 10^8^ inclusion-forming units per animal. Six calves were inoculated with the same dose of inactivated *Cp* propagated in BGM cells as well. Inactivation was achieved by exposure to UV, and was confirmed in cell culture passages as described previously [18]. Twenty one calves were inoculated with BGM cells only. Three naïve sentinels were co-housed with the group inoculated with vital *Cp* on 2 dpi. For further details of this bovine model of respiratory *Chlamydia psittaci* infection, see [18–21].

**Fig 1:**
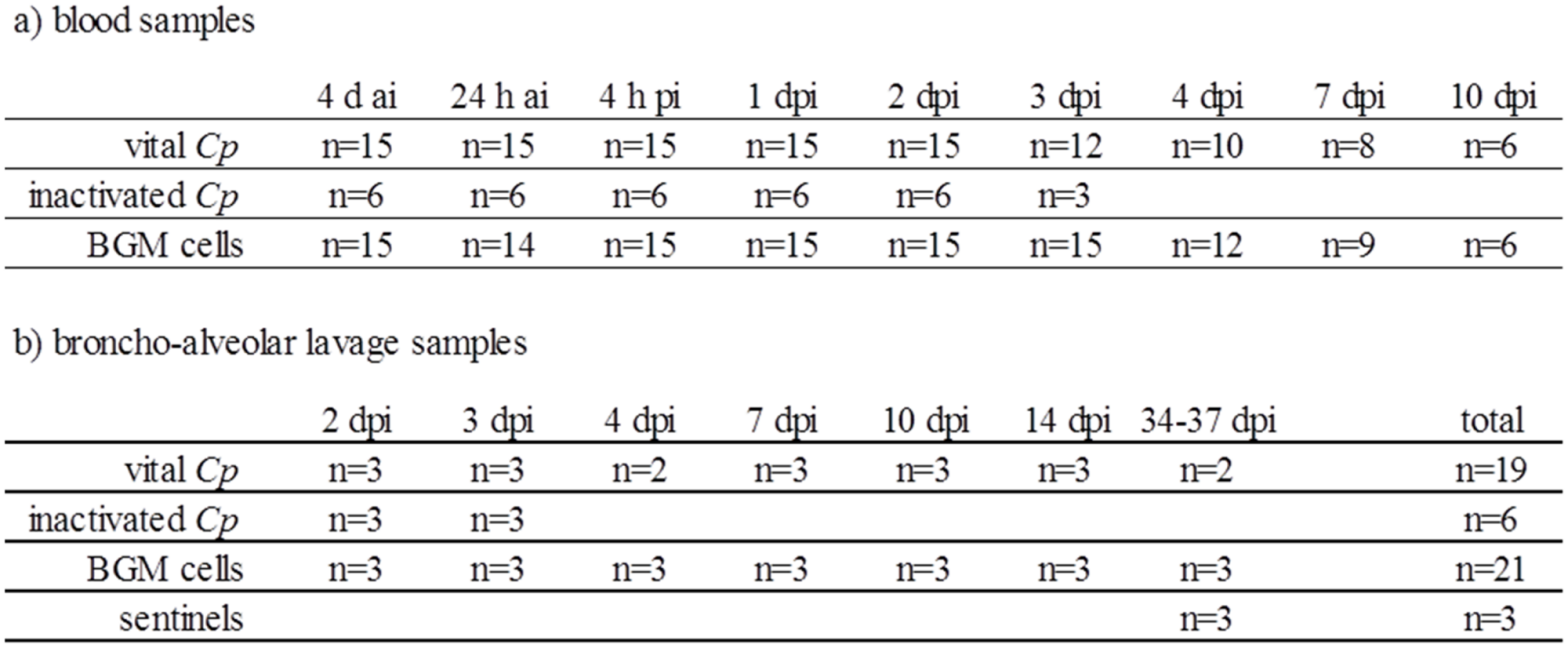
Study design. d ai/h ai = days/hours ante inoculation; dpi = days post inoculation.

Blood was sampled from 15 animals from each of the groups inoculated with vital *Cp* and BGM cells, and from the six calves inoculated with inactivated *Cp*. For time points and number of sampled animals, see Fig 1a. Rectal body temperature was measured every morning until 14 dpi in all animals. In the six calves inoculated with inactivated *Cp*, the abdominal aorta was catheterized the day before challenge to collect arterial blood for another study [19]. Animals from all groups subsequently underwent necropsy to sample BALF, in groups of two to three animals per time point. Time points of necropsy and number of animals necropsied are given in Fig 1b. Animals were sacrificed by exsanguination under conditions of deep anesthesia (method of euthanization has been described in detail elsewhere [18]).

### Measurement of IL-6 in blood and broncho-alveolar lavage fluid

Venous blood was collected from the jugular vein before morning feeding using 9.0 mL plastic syringes (S-Monovette, Sarstedt). Serum was harvested by centrifugation and stored at -80°C until analysed.

Broncho-alveolar lavage fluid (BALF) was obtained from freshly exenterated lungs immediately after exsanguination, as described previously [18]. BALF supernatant was obtained by immediate centrifugation (300 x *g*; 20 min), and was stored at -80°C until analysis.

IL-6 was determined in blood serum or BALF supernatant, respectively, by a proliferation assay on the IL-6-dependent 7TD1 hybridoma cell line. This cell line has previously been used to measure IL-6 in plasma of cows [22]. The assay was performed in sterile, 96-well microtiter plates. In each well, 5000 7TD1 cells were incubated for 72 h with serial dilutions of biological samples or with different concentrations of a human IL-6 standard (code 89/548, National Institute for Biological Standards and Control, South Mimms, U.K.). Samples were pre-diluted so that serial dilution of samples and standard dilution curves were parallel. The number of living cells after 72 h was measured using the dimethylthiazol-diphenyl tetrazolium bromide (MTT) colorimetric assay [23].

### Statistical methods

R [24] and Statgraphics Centurion XVI (version 16.1.18, Statpoint Technologies) were used for statistical evaluation. Since data were not normally distributed (Shapiro-Wilk test), only non-parametric tests were chosen for statistical evaluation. Intra-individual comparisons were performed for IL-6 activities in blood and corresponding rectal temperature. The Wilcoxon signed rank test with zero handling according to Pratt from the coin package [25] was used along with Holm adjustment to compare pre- with post-inoculation values. IL-6 activities in BALF were only available at necropsy for each animal. Therefore, inter-individual comparison was carried out here and also with the corresponding rectal temperature. To compare the different challenge groups, the two-sided Mann-Whitney U test with Holm adjustment was used. To compare activities of IL-6 between blood and BALF, the sign test (comparing medians) and Spearman’s rank correlation analysis were applied. Multiple regression analysis was exploited to identify significant relationships between IL-6 and other markers of inflammation (i.e., inflammatory cells, eicosanoids) assessed in parallel within blood or BALF, respectively.

Values of *P* ≤ 0.05 were considered significant. Values of 0.05 ≤ *P* < 0.1 were regarded as trends and are given in the graphs. In box and whisker plots, outlier values (circles) are more than 1.5 times of the length of a box away from the median. For *n* = 3, no statistical analysis was carried out and data are given as dots.

## Results

### Physiological IL-6 activities in peripheral blood

Table 1 shows IL-6 activities assessed in the peripheral blood of healthy calves aged 6–8 weeks. Catheterized animals exhibited significantly higher IL-6 activity (> 200 I.U./mL blood) compared to non-catheterized calves, which exhibited IL-6 activities less than 100 I.U./mL blood on average (Day 1: *P* = 0.001; Day 2: *P* = 0.0004, Mann-Whitney U test). For these two groups, there was no significant difference between the two sampling days within one week (Wilcoxon test, *P* > 0.05), and intra-subject variability on two consecutive days was less than 20%, on average. On the group level, data were not normally distributed (Shapiro test, *P* < 0.05), and inter-subject variability ranged between 54% and 80%.

**Table 1:** Activity of IL-6 (I.U./mL) in blood serum of healthy calves aged 6–8 weeks at two different days within one week.

	*time*	*n*	*average*	*median*	*standard deviation*	*minimum*	*maximum*	*range*	*CV intra-subject*	*CV inter-subject*
non-catheterized	Day 1	30	72.8	57.0^a^	58.5	14.0	273.0	259.0		80.4%
	Day 2	29^#^	69.1	69.0^a^	37.1	9.0	141.0	132.0		53.7%
	Total	59^#^	71.0	63.0	48.8	9.0	273.0	264.0	18.7% (0.7–54.1)	68.7%
catheterized	Day 1	6	241.3	222.0^b^	139,1	126.0	507,0	381,0		57,6%
	Day 2	6	241.5	204.5^b^	145.2	98.0	520,0	422,0		60.1%
	Total	12	241.4	209.5	135.5	98.0	520,0	422,0	14.1% (1.2–37.0)	56.2%

^#^ one missing value

^a,b^ different letters indicate significant differences between groups, *P* < 0.01 (Mann-Whitney-U test)

*CV intra-subject*: individual coefficient of variability for IL-6 between two different days; given as mean (min—max) for each group

*CV inter-subject*: coefficient of variability within each group per day

### IL-6 in blood serum of inoculated animals

**Fig 2:**
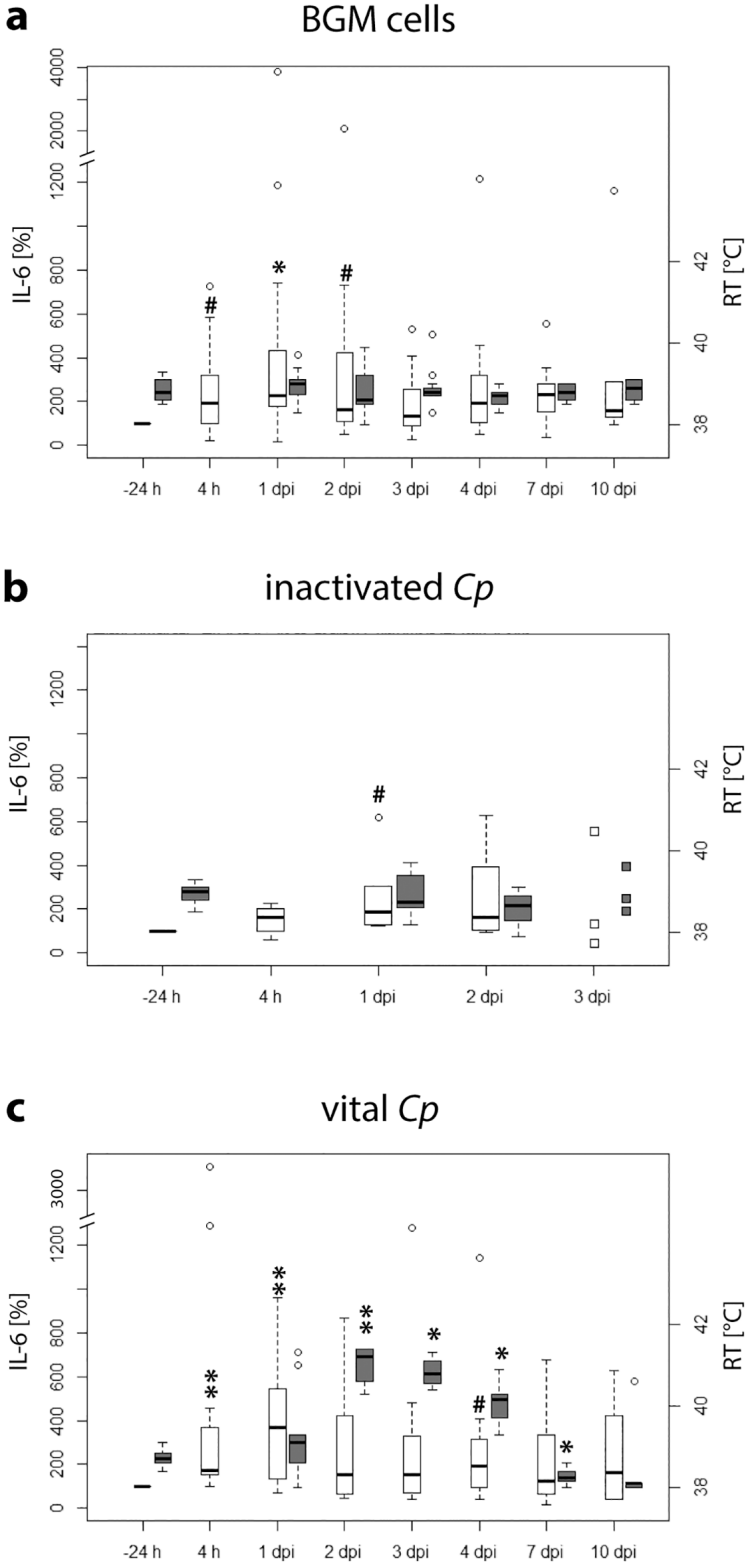
Relative changes of IL-6 in blood serum of calves and rectal body temperature in different treatment groups. Relative activity of IL-6 in blood serum (white) and rectal body temperature (RT) (grey) are presented box and whisker plots for *n* ≥ 6 and as plots for *n* = 3. *Cp*: *Chlamydia psittaci*. x-axis: relative changes of IL-6 in blood serum in % and rectal body temperature in °C. y-axis: time. -24 h: 24 hours before inoculation; 4 h: 4 hours after inoculation. Post-inoculation values of animals were compared to pre-inoculation values of the same animals with the Wilcoxon signed rank test, then *P*-values were adjusted according to Holm (# 0.05 < *P* ≤ 0.1; * 0.01 < *P* ≤ 0.05; ** 0.001 < *P* ≤ 0.01).

The activity of IL-6 in blood serum of calves was increased compared to pre-inoculation values in all groups (Fig 2). In BGM-inoculated animals, the increase of IL-6 in blood serum was significant at 4 h, 1 dpi, and 2 dpi. In the group inoculated with inactivated *C*. *psittaci*, there was a tendency for blood serum IL-6 to increase at 1 dpi. Animals inoculated with vital *C*. *psittaci* showed significantly increased IL-6 levels in the blood serum at 4 h and 1 dpi. Pre-inoculational activities of IL-6 were not reached in any of the groups by the end of the study.

### IL-6 in BALF of inoculated animals

**Fig 3:**
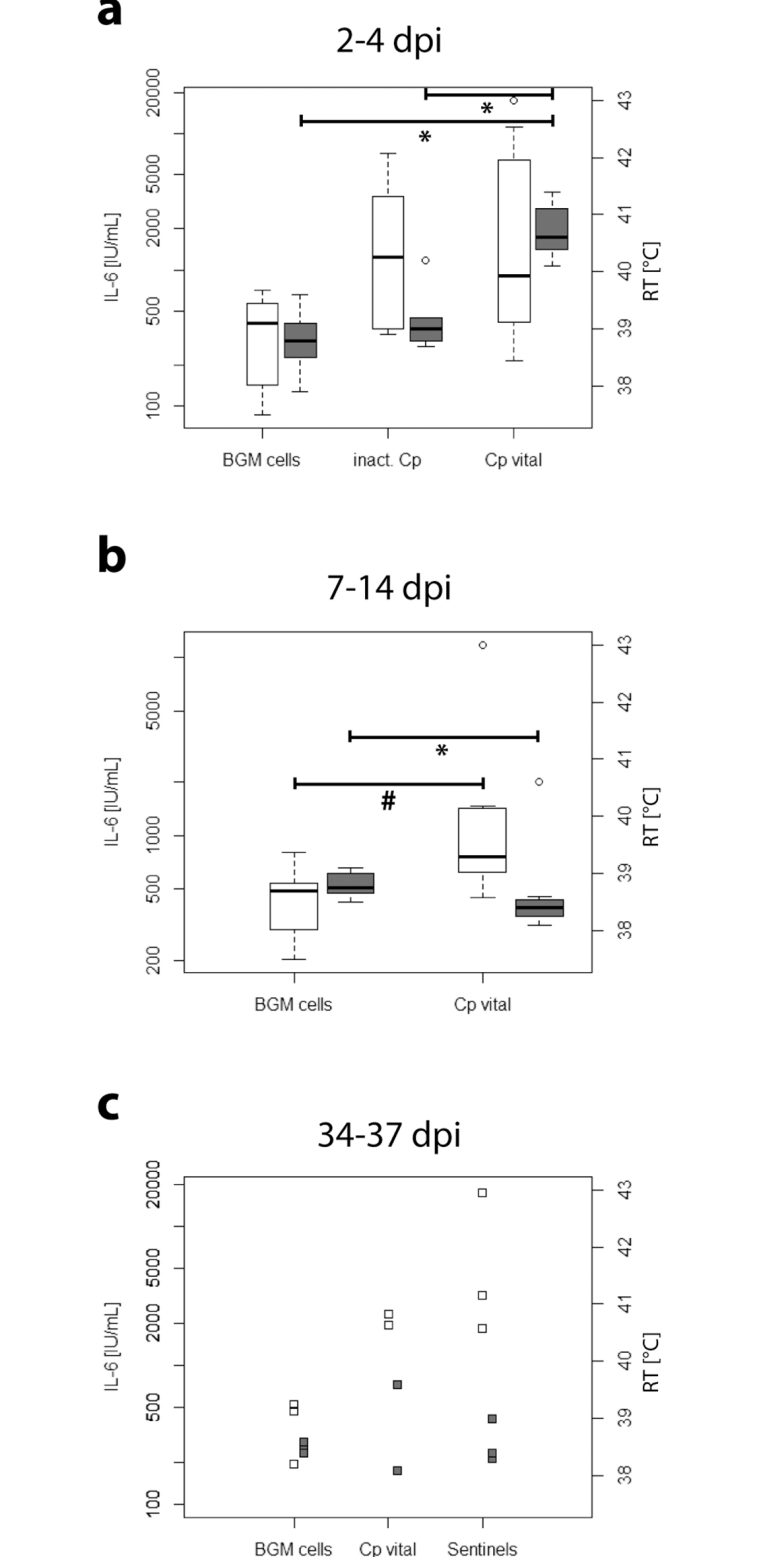
Activity of IL-6 in bronchoalveolar lavage fluid and rectal body temperature of calves of different treatment groups at different time points after inoculation. Values of the different treatment groups were compared with the Mann-Whitney-U-Test (# 0.05 < *P* ≤ 0.1; * 0.01 < *P* ≤ 0.05). No statistical analysis was carried out for 34–37 dpi due to small sample sizes (*n* = 2–3 per group). dpi: days post inoculation, RT: rectal body temperature, Cp vital: vital *Chlamydia psittaci*, inact. Cp: inactivated *Chlamydia psittaci*.

Animals inoculated with vital or inactivated *Cp* had higher IL-6 activity in their BALF than animals inoculated with BGM cells (Fig 3). During the acute phase of the disease from 2–4 dpi this was not significant, whereas a tendency was found during the recovery phase comparing animals inoculated with vital *Cp* to animals inoculated with BGM cells. Interestingly, this increase was still visible more than a month after inoculation. Animals inoculated with vital *Cp* and animals that acquired the infection naturally had higher amounts of IL-6 in their lavage than animals inoculated with BGM cells.

### Comparison of IL-6 activities between BALF and blood

When IL-6 was assessed in BALF and in blood serum (on the same day in the same animal; i.e., after challenge on the day of necropsy) absolute IL-6 activity was significantly higher in BALF compared to that in blood (Table 2). No significant rank correlations existed between the IL-6 activities measured at the two different body sites in any group of calves.

**Table 2:** Activities of IL-6 (I.U./mL) in BALF and blood serum assessed at the same day in calves.

*challenge*	*IL-6 [I*.*U*.*/mL]*	*n*	*average*	*median*	*standard deviation*	*minimum*	*maximum*	*range*	*sign test*
vital *Cp*	BALF	14	2012.7	672.0	4509.1	213.0	17596.0	17383.0	*P* < 0.001
	Blood serum	14	171.5	110.5	149.3	9.0	519.0	510.0	
inactivated *Cp*	BALF	6	2289.5	1245.0	2660.1	334.9	7112.0	6778.0	n.s. (*P* = 0.68)
	Blood serum	6	523.5	449.0	380.6	114.0	979.0	865.0	
BGM cells	BALF	15	460.1	491.0	210.1	86.0	803.0	717.0	*P* < 0.002
	Blood serum	15	150.1	79.0	157.4	20.0	530.0	510.0	
**Total**	BALF	35	1394.8	583,0	3084.9	86.0	17596.0	17510.0	***P* = 0.0000**
	Blood serum	35	222.7	118.0	243.7	9.0	979.0	970.0	

The sign test tests the null hypothesis that the median IL-6 (BALF)-IL-6 (blood serum) equals 0.0 versus the alternative hypothesis that the median IL-6 (BALF)-IL-6 (blood serum) is not equal to 0.0. It is based on counting the number of values above and below the hypothesized median. Since the *P*-value for this test is less than 0.05, we can reject the null hypothesis at the 95.0% confidence level.

### Relations between IL-6 and rectal body temperature

Rectal body temperature only increased after inoculation in the group challenged with vital *C*. *psittaci*. This increase was significant from 2 dpi until 4 dpi. Interestingly, the maximal increase in body temperature at 2 dpi was one day after the maximal increase of IL-6 in blood serum. Rectal body temperature of the animals inoculated with vital *Cp* was within physiological limits from 7 dpi onwards, being significantly lower than before inoculation (Fig 2) and significantly lower than in animals inoculated with BGM cells (Fig 3).

### No relations between IL-6 and blood composition

Within blood, multiple regression analysis did not reveal any significant relationship between IL-6 activity and number of leucocytes, differentiated white blood cells, number of erythrocytes, haematocrit, or concentration of haemoglobin in any group or at any time point (*n* = 262 samples of 35 calves; data not shown).

### Existing relations between IL-6 and markers of inflammation in broncho-alveolar lavage fluid

Including all data assessed in BALF in a multiple linear regression analysis, results of the best fitting model revealed the existence of significant relationships between IL-6 activity and the following four variables: number of lymphocytes per mL and concentrations of thromboxane B_2_ (TXB_2_), prostaglandin E_2_ (PGE_2_), and lipoxin A_4_ (LxA_4_) (Table 3; absolute data not shown).

**Table 3:** Results of multiple regression analysis relating IL-6 to other markers of inflammation in broncho-alveolar lavage fluid in all inoculated calves.

dependent variable: IL-6 [IU/ml]				
independent variables: Ly, TXB_2_, PGE_2_, LxA_4_				
*parameter*	*estimate*	*standard error*	*t statistic*	*P-value*
CONSTANT	6266.41	1180.31	5.30913	0.0000
lymphocytes per mL (Ly)	-0.0712757	0.0238974	-2.98258	0.0047
TXB_2_ [ng/ml]	-282.988	124.677	-2.26978	0.0283
PGE_2_ [ng/ml]	-21477.7	5324.26	-4.03393	0.0002
LxA_4_ [ng/ml]	21854.5	4864.03	4.49308	0.0001
equation of the fitted model: *IL-6 [IU/mL] = 6266*.*41 − 0*.0713*lymphocytes/mL − 282.988*TXB_*2*_ *[ng/mL] − 21477*.7*PGE_*2*_ *[ng/mL] + 21854*.5*LxA_*4*_ *[ng/mL]*.				

The R-squared statistic indicates that the model as fitted explains 35% of the variability in IL-6 (*R*-squared = 34.96 percent; *P* = 0.0008, *n* = 48).

Repeating this multiple linear regression analysis for the three groups separately, the significant relationships between IL-6 and lymphocytes, PGE_2_, and LxA_4_ were confirmed for those calves that had been challenged with vital chlamydiae (Table 4). The two groups inoculated with either inactivated *Cp* or BGM cells did not show significant relationships between IL-6 and potential markers of inflammation assessed in BALF (data not shown).

**Table 4:** Results of multiple regression analysis relating IL-6 to other markers of inflammation in broncho-alveolar lavage fluid in calves inoculated with 10^8^ ifu of vital *Chlamydia psittaci*.

dependent variable: IL-6 [IU/ml]				
independent variables: Ly, TXB_2_, PGE_2_, LxA_4_				
*parameter*	*estimate*	*standard error*	*t statistic*	*P-value*
CONSTANT	10679.2	2909.77	3.67012	0.0028
lymphocytes per mL (Ly)	-0.133702	0.0449465	-2.97469	0.0107
TXB_2_ [ng/ml]	-319.974	381.937	-0.837767	0.4173
PGE_2_ [ng/ml]	-33186.8	11401.6	-2.9107	0.0122
LxA_4_ [ng/ml]	33099.7	11384.4	2.90745	0.0122
equation of the fitted model: *IL-6 [IU/mL] = 10679*.*2 − 0*.133702*lymphocytes/mL − 319.974*TXB_*2*_ *[ng/mL] − 33186*.8*PGE_*2*_ *[ng/mL] + 33099*.7*LxA_*4*_ *[ng/mL]*.				

The R-squared statistic indicates that the model as fitted explains 53% of the variability in IL-6 (*R*-squared = 53.19 percent; *P* = 0.0321, *n* = 18).

No relationships existed between IL-6 activity and numbers of alveolar macrophages, granulocytes, or total cell number per mL BALF, respectively, or between IL-6 activity and concentration of protein in BALF in any group (data not shown).

## Discussion

One central goal of this study was to assess a possible relationship between circulating IL-6, local formation of IL-6 within stimulated lung tissue, and fever in response to an intra-bronchial challenge with vital *Cp*, inactivated *Cp*, or with BGM cells. Storage temperature seems to play an important role in IL-6 stability in samples. Samples analysed in this study were stored at -80°C, whereas samples from another trial were stored at -20°C. The latter could not be analysed successfully (data not shown). We therefore recommend storing IL-6 samples at -80°C.

Independent from the intra-bronchial administration of the distinct compounds, we detected that catheterization of the abdominal aorta causes the appearance of significantly higher levels of bioactive IL-6 in the blood when compared to non-catheterized calves (Table 1). IL-6 is a pleiotropic cytokine, which is synthetized not only by immune cells but also by various other cell types, including muscle tissue or endothelial cells [26, 27]. All these cells can be activated by so-called “pathogen-associated molecular patterns” (PAMPs) via pathogen recognition receptors. As a consequence, a pro-inflammatory cytokine cascade is induced in the infected host, with IL-6 being one of the most prominent members ([1], for review). Pathogens are, however, not the exclusive triggers for the formation of cytokines. Trauma and tissue injury cause the release of endogenous, usually intracellular, molecules named “alarmins” or “damage-associated molecular patterns” (DAMPs, [28]). These molecules also activate pathogen recognition receptors in the absence of pathogens and thereby induce cytokines, namely IL-6. Indeed, circulating IL-6 has been proposed as a sensitive marker of trauma or injury in humans [29]. IL-6 is even elevated in the blood during psychological stress [30, 31] or after prolonged exercise [32]. It is thus likely that the significantly elevated levels of IL-6 in the blood of catheterized calves were caused by the surgical implantation of the catheter. The injury induced by the catheter could readily have caused release of DAMPs and thereby activation of endothelial cells in the catheterized vessel.

A striking result of this study was that IL-6 in blood serum was significantly elevated not only after inoculation with vital *Cp* but also with BGM cells. A reasonable explanation is that administering BGM cells into the lung caused similar tissue irritation to placement of the catheter (see above) and thereby release of IL-6 into the blood. It is, however, difficult to explain, why there is a stronger elevation of IL-6 in the blood of calves, treated with BGM cells compared to animals occulated with inactivated *Cp*, since the concentrations of IL-6 in BALF of calves treated with inactivated or vital *Cp* are substantially higher compared to the group treated with BGM cells. This discrepancy may be due to rather large variations of IL-6 in serum or BALF under distinct inflammatory conditions. Surprisingly, the increased circulating levels of IL-6 were only accompanied by manifestation of fever in calves inoculated with vital *Cp*, while treatment with BGM cells did not cause any significant elevation of body temperature in spite of the enhanced concentrations of IL-6 in the blood. This result challenges the frequently proposed role of IL-6 as a critical circulating endogenous pyrogen [5–7, 10], although it should be stated that a lack of correlation between circulating IL-6 and the manifestation of fever does not exclude the proposed role for IL-6 as a critical circulating fever-inducing agent.

Assessment of fever started 24 hours (1dpi) after inoculation in this study because previous observations (when the model was established and evaluated) revealed that no significant increase in rectal temperature was observable in any group 4 or 12 hours after challenge [18, 21]. Nevertheless, one might speculate that we missed a short and early phase of fever and / or an early rise of circulating IL-6 within the first 24 hours even in the group treated with BGM cells. It is known that fever frequently consists of several phases (early and late phases; see [1] for review). Based on the lack of data during this time interval it remains speculative to consider a better correlation between circulating IL-6 and body temperature changes during this early interval.

It should also be noted that all experimental studies which suggested a role for IL-6 as an obligatory circulating pyrogen were performed with LPS as a fever-inducing agent. Even in these studies it had to be admitted that IL-6 cannot be the only endogenous pyrogen, due to the rather moderate pyrogenic effect of its exogenous administration [6, 11]. The situation in experimentally simulated real infections is even more complex. Several studies which aimed to mimic real infection found no correlation between the manifestation of febrile episodes and the appearance of IL-6 in the blood [12, 13]. It is likely that *Cp* caused the induction of additional mediators of inflammation, namely at the site of inoculation (Table 4), which accounted for the manifestation of fever.

In this context, it must be considered that fever and other brain-controlled signs of illness can be initiated by distinct immune-to-brain communication pathways. Under conditions of very high levels of pro-inflammatory cytokines, including IL-6, in the blood (“cytokine cascade”), fever will be induced by direct action of these endogenous pyrogens on the brain via interactions with endothelial cells or by cytokines entering the brain parenchyma at sites with an incomplete blood-brain barrier [33]. The concentrations of IL-6 which we measured in the blood of calves inoculated with vital *Cp* or with BGM cells were substantially lower than those detected during systemic inflammation induced by LPS-injections [7, 11]. As a consequence, we have to consider one of the alternative signalling pathways which can transfer fever-inducing signals into the brain. In experimental models which mimic localized subcutaneous inflammation by injections of LPS or other PAMPs into a subcutaneous chamber or an air pouch, very high levels of IL-6 can be measured in the lavage collected from the subcutaneous site of inflammation, while the levels of IL-6 in the blood only increase slightly [14]. However, fever is still observed under these conditions, and there is some evidence that the activation of afferent nerve fibres by inflammatory mediators plays a role in generating a febrile response under these conditions. Specifically, PGE_2_ is a likely candidate for stimulating such a neuronal afferent immune-to-brain communication pathway [34–36]. Some parallels to our present study in calves can be shown. First, there are significantly higher levels of IL-6 in broncho-alveolar lavage fluid (BALF) compared to those in blood serum (Table 2). Second, there seems to be a relationship between IL-6 and other mediators of inflammation, including PGE_2_, in BALF of inoculated calves. Interestingly, calculations performed for the three groups separately (Table 4) showed that this relationship between IL-6 and other mediators including PGE_2_ remains manifest only in the group, which developed fever (challenge with vital *Cp*). PGE_2_ is known to stimulate afferent fibres of the hepatic branch of the vagus nerve and thereby contributes to the manifestation of fever [1]. A similar mechanism could operate in the lungs of inoculated calves: a localized formation of IL-6, PGE_2_ and possibly other mediators might stimulate afferent vagus fibres originating in the lung tissue and thereby evoke the increase in rectal temperature more or less independently from the circulating concentrations of IL-6. A case in point against this argument is given by the data shown in Table 2. Concentrations of IL-6 in BALF were almost identical in calves treated with vital or inactivated *Cp*, while fever (at the investigated time intervals) only occurred in animals suffering from the real infection (Fig 2). In this context it seems worthwhile to take a look at the pathomechanisms, which are employed by the host infected with vital *Cp* [37]. The host’s successful combat against chlamydial infections requires activation of both, innate and adaptive immune responses and complex interactions between both of them [37]. In mice infected with vital *Cp* in the lung a long-lasting activation of the complement system was observed [38]. Especially complement fragment C3 seems to be critical for a successful combat against this intracellular microorganism. Infected mice deficient in C3 showed a 100% mortality, while there was an almost complete survival in wild-type mice with *Cp* lung infection due to stimulating effects of C3 on adaptive immunity [38, 39]. Interestingly, complement is also an essential component for the manifestation of fever in some experimental fever models [1, 16, 17]. There is evidence that activation of the complement cascade by bacterial lipopolysaccharide (LPS) leads to formation of PGE_2_ by Kupffer cells, which are macrophages of the liver [16, 17]. PGE_2_, in turn, has the capacity to stimulate afferent fibres of the vagus nerve to induce fever (see above). A mechanism like this might operate in the lung of vital *Cp* infected calves. The lack of fever the other groups (inactivated *Cp*, BGM cells), at the investigated time intervals, might thus be due to the missing activation of the complement system and thereby a lack of sufficient amounts of PGE_2_. To prove this hypothesis, one would have to induce a hypo-complementation by cobra venom factor [16] or to interrupt the afferent neuronal connection between the infected lung and the brain and test whether the febrile response is blunted under these conditions. Still, the outcome from the multiple regression analysis, showing that only in BALF of calves challenged with vital *Cp* a significant relationship between locally produced IL-6 and other markers of inflammation could be determined, supports such an interpretation of our data.

## Conclusions

In conclusion, the fever which we observed in calves after administering *Cp* into the lungs was caused by a locally generated inflammatory cascade rather than circulating IL-6. Increased IL-6 activity alone is not necessarily associated with fever induction, but a participation of IL-6 in the manifestation of a febrile response during vital *Cp* infections cannot be excluded.
